# Core Prescribing Indicators and the Most Commonly Prescribed Medicines in a Tertiary Health Care Setting in a Developing Country

**DOI:** 10.1155/2021/6625377

**Published:** 2021-01-29

**Authors:** Priyadarshani Galappatthy, Priyanga Ranasinghe, Chiranthi K. Liyanage, Maheshi Wijayabandara, Dinuka S. Warapitiya, Dilini Jayasekara, Raveendra L. Jayakody

**Affiliations:** ^1^Department of Pharmacology, Faculty of Medicine, University of Colombo, Colombo, Sri Lanka; ^2^Colombo North Teaching Hospital, Ragama, Sri Lanka

## Abstract

Irrational prescribing is common, especially in developing countries. It is important to identify the magnitude of irrational use, to take necessary steps to promote rational prescribing. We identified core prescribing indicators and commonly prescribed medicines at ward settings (IW) and outpatients' clinics (OPC) in a tertiary care hospital in Sri Lanka. A descriptive cross-sectional study was carried out at IW and OPC settings. Prescriptions were obtained from 5 major specialties (Clinical Medicine (CM), Gynaecology and Obstetrics (GO), Paediatrics, Psychiatry, and Surgery). The WHO core prescribing indicators were used to describe the pattern of prescribing, and the most commonly prescribed medicines were identified. A total of 1,318 prescriptions were analyzed. The five most commonly prescribed medicines were paracetamol (31.0%), omeprazole (20.6%), folic acid (18.3%), atorvastatin (16.2%), and salbutamol (15.3%). The average number of medicines per encounter was 4.8 ± 3.6 (IW: 5.7 ± 4; OPC: 3.8 ± 2.8; *p* < 0.001), with the highest IW (7.8 ± 4.2) and OPC (7.8 ± 2.7) values were from CM, being significantly higher than all other disciplines (*p* < 0.05). Percentage encounters with an antibiotic or an injection was 26.4% and 30.1%, respectively, with IW being significantly higher than OPC (*p* < 0.001). Percentage of medicines prescribed by generic name and from the essential medicine list (EML) was 90.1% and 91.1%, respectively, with no significant IW and OPC difference. In conclusion, a high degree of polypharmacy was noted. The use of injectable medicines, prescribing from the EML, and generic name prescribing was satisfactory; however, overall rational prescribing needs further improvement. Further investigation into the degree of rational prescribing associating it with clinical information will be important.

## 1. Introduction

The World Health Organization (WHO) defines rational use of medicines as prescribing the right medicine, for the right patient, at the right dose, for the right duration, and at the right (lowest) cost to them and their community [1]. However, studies have shown that more than 50% of all medicines are prescribed, dispensed, or sold inappropriately, with a similar percentage of patients failing to take their medicines correctly [2]. The most common types of irrational use of medicines include polypharmacy, inappropriate use of antimicrobials, overuse of injectable preparations, prescription of medicines without adhering to clinical guidelines, and inappropriate self-medication [2]. Irrational use of medicines leads to several issues; for example, polypharmacy in the elderly is associated with increased rates of adverse drug reactions, interactions, and nonadherence to medications [3]. In addition, misuse of antimicrobials is a key causative factor accelerating the emergence of antimicrobial resistance [4]. Furthermore, irrational use of medicines also contributes to a substantial increase in cost to health care systems around the world [5]. Hence, it is important to identify the magnitude of irrational use of medicines, in order to take necessary steps to promote rational prescribing. For this purpose, the WHO in collaboration with the International Network of Rational Use of Drugs (INRUD) has developed core prescribing indicators to evaluate practices of medicine usage in health care settings [6].

The problem of irrational medicine use is known to be worse in developing countries with weak health systems, where mechanisms for routine monitoring of medicine use are often not well developed [7]. Sri Lanka is a rapidly developing island nation in the South Asian region that has a population of nearly 22 million [8]. Health care services in the country are mostly provided through a network of government-operated public health care services at different levels. Sri Lanka is recognized internationally for its good health indicators at a quite low level of gross domestic product (GDP) and is at the forefront in the South Asian region in providing quality health services [9]. A study conducted in outpatient clinics in a teaching hospital in the Galle district of Sri Lanka revealed that the average number of medicines per prescription was 3.24 with 47% of the prescriptions containing antibiotics [10]. However, there are no recent studies exploring the problem of irrational medicine use in Sri Lanka, especially from tertiary care hospitals. The commercial capital and economic hub of Sri Lanka is situated in Colombo, which also has the country's largest tertiary care referral centers in the five major disciplines—Clinical Medicine, Surgery, Gynaecology and Obstetrics, Paediatrics, and Psychiatry. At present, there are no studies evaluating the practices of medication use in this group of tertiary care hospitals in Sri Lanka. Furthermore, since these hospitals also function as teaching hospitals for medical undergraduates and postgraduates, understanding and strengthening rational use of medicines in these hospitals is important. The purpose of this study was to identify the core prescribing indicators and commonly prescribed medicines at ward settings and outpatients' clinics in the Colombo group of teaching hospitals in the five major specialties. The findings of this study would help the policy makers to take appropriate actions to promote rational use of medicines in Sri Lanka.

## 2. Materials and Methods

### 2.1. Study Setting

The study was carried out at the wards and outpatient's clinics in the Colombo group of tertiary care teaching hospitals namely, National Hospital of Sri Lanka (NHSL), Lady Ridgeway Hospital (LRH) for Children, and De Soysa Hospital for Women (DSHW) between March and August in 2015. The NHSL is the largest hospital in Sri Lanka and South East Asia with a bed strength of nearly 3500, while the LRH is a specialized tertiary care hospital for children with a bed strength more than 1000 and considered to be the largest children's hospital in the world. The DSHW is a specialized tertiary care hospital for women and has a bed strength of around 500. All prescriptions are written manually in all settings included in the present study. Ethical approval for the study was obtained from the Ethics Review Committee, Faculty of Medicine, University of Colombo (ERC-14-021), and institutional approval was obtained from the NSHL, LRH, and DSHW.

### 2.2. Study Design, Definitions, and Outcome Measures

A descriptive cross-sectional study was designed to identify core prescribing indicators and commonly prescribed medicines. According to WHO recommendations, at least 600 encounters should be included in a cross-sectional survey in order to describe the prescribing indicators [6]. Prescriptions were obtained from five major specialties, namely Clinical Medicine (CM), Gynaecology and Obstetrics, Paediatrics, Psychiatry, and Surgery. Data on prescriptions were collected from both in-ward and outpatient clinic settings simultaneously. In-ward prescriptions were obtained from the University Wards of the NHSL (Clinical Medicine: Ward 41/48B; Psychiatry: Ward 59), LRH (Paediatrics: Ward 1), and DSHW (Gynaecology and Obstetrics: Ward 7/15), where 160 consecutive prescriptions (80 in-ward and 80 outpatient clinic) from each of the above units were obtained (except Psychiatry). Furthermore, in order to have a representative sample of prescriptions from each discipline, two other randomly selected units from each discipline were included; and from these units, 80 consecutive prescriptions each were analyzed (40 in-ward and 40 outpatient clinic). Hence, from each discipline (except Psychiatry), 320 prescriptions were planned to be analyzed (160 in-ward and 160 outpatient clinic); however, as there is only one psychiatry unit situated at NHSL (Ward 59), only 80 prescriptions were collected in that discipline (40 in-ward and 40 outpatient clinic). Hence, we planned to collect a total of 1360 prescriptions (680 in-ward and 680 outpatient clinic) for the present analysis.

The WHO core prescribing indicators listed below were used in our study to describe the pattern of prescription [6]:

  (1) Average number of medicines per encounter—calculated by dividing the total number of different medicines prescribed by the number of prescriptions surveyed (WHO recommended value, 1.6–1.8)
  (2) Percentage of encounters with an antibiotic—calculated by dividing the number of encounters in which an antibiotic was prescribed by the total number of encounters surveyed, multiplied by 100 (WHO recommended value, 20–26.8%)
  (3) Percentage of encounters with an injection—calculated by dividing the number of encounters in which an injection was prescribed by the total number of encounters surveyed, multiplied by 100 (WHO recommended value, 13.4–24.1%) (Injectables were defined as any medicine injected via intravenous, intramuscular, subcutaneous, or other parenteral routes of administration)
  (4) Percentage of medicines prescribed by generic name—calculated by dividing the number of medicines prescribed by generic name by total number of medicines prescribed, multiplied by 100 (WHO recommended value, 100%)
  (5) Percentage of medicines prescribed from the essential medicine list—calculated by dividing number of medicines prescribed that are in the essential medicine list [11] by the total number of medicines prescribed, multiplied by 100 (WHO recommended value, 100%)

The above prescribing indicators were evaluated in comparison to WHO recommended optimal values as shown above [6].

Zhang and Zhi developed an index system for the comprehensive evaluation of health care systems [12]. For the calculation of indices of non-polypharmacy, rational antibiotic use, and safe injection use, the WHO optimal value was divided by the observed value. To obtain the indices of generic name and medicines from the essential medicines list, the observed value was divided by the WHO optimal value. The optimal index for all indicators was set as 1, where values closer to 1 indicated rational use. The Index of Rational Drug Prescribing (IRDP) was calculated for the different disciplines by adding the index values of all prescribing indicators.

### 2.3. Data Collection and Analysis

Data were collected by three trained medical graduates from bed head tickets, prescription charts, and clinic records. Data were collected at different times during the data collection period (March–August, 2015), and for outpatients, data depended on clinic dates of the respective disciplines. Data were recorded in a self-designed form formulated by modifying the WHO ordinary form for prescribing indicators [6]. Reliability of the data was ensured by following the WHO guidelines and methods [6]. Data from a single sampling unit were collected by the same data collector to avoid duplication of data. In addition to the WHO prescribing indicators, the most commonly prescribed medicines were also analyzed. Statistical Package for Social Sciences (IBM SPSS Statistics for Windows, version 14.0; IBM Corp., Armonk, NY) was used for analysis of data. Descriptive statistics such as frequencies, percentages, and mean and standard deviation were measured. Differences among the health care facilities were established using ANOVA tests. The manuscript reporting adheres to STROBE guidelines (Supplementary File 1). The statistical significance was determined by a *p* value < 0.05.

## 3. Results

A total of 1,318 prescriptions were analyzed (96.9%), and 42 prescriptions with incomplete records were excluded from analysis. The number of prescriptions from each discipline (in-ward (IW)/out-patient clinic (OPC)) was as follows—Medicine: 160/160, Gynaecology and Obstetrics: 160/160, Paediatrics: 118/160, Psychiatry: 40/40, and Surgery: 160/160. Overall, the ten most commonly prescribed medicines were paracetamol (*n* = 409; 31.0%), omeprazole (*n* = 272; 20.6%), folic acid (*n* = 241; 18.3%), atorvastatin (*n* = 213; 16.2%), salbutamol (*n* = 202; 15.3%), ferrous sulphate (*n* = 179; 13.6%), ascorbic acid (*n* = 171; 13.0%), calcium lactate (*n* = 166; 12.6%), domperidone (*n* = 159; 12.1%), and aspirin (*n* = 150; 11.4%).  Table 1 shows the 10 most commonly prescribed medicines in each of the disciplines. The commonest antibiotic, antiepileptic, antidiabetic, and antihypertensive prescribed were metronidazole (*n* = 121; 9.2%), sodium valproate (*n* = 66; 5%), metformin (*n* = 143; 10.8%), and losartan (*n* = 114; 8.6%), respectively. The 100 most prescribed medicines overall (Supplementary File 2) and in each discipline (Supplementary File 3) are included as Supplementary Material.

### 3.1. Medicines per Encounter

A total of 6,280 medicines were prescribed (IW/OPC: 3,663/2,617) in all the prescriptions, with the highest number being from Clinical Medicine (2,498; IW/OPC: 1,247/1,251), followed by Surgery (1,446; 1,121/325), and Gynaecology and Obstetrics (1,194; 683/511). The combined average number of medicines per encounter in all disciplines was 4.8 ± 3.6 (median: 4) with a range between 1 and 22 (IW: 5.7 ± 4; OPC: 3.8 ± 2.8) (Table 2). It was significantly higher for IW prescriptions in the overall analysis (*p* < 0.001). The highest average number of medicines per encounter IW (7.8 ± 4.2), OPC (7.8 ± 2.7), and combined (7.8 ± 3.8) was from Clinical Medicine, which was significantly higher than all other disciplines in the combined analysis of both IW and OPC (Table 2). The lowest average number of IW (3.1 ± 2.6) and OPC (2.0 ± 1.0) medicines per encounter was from disciplines of Paediatrics and Surgery, respectively, being significantly lower than respective averages from all other disciplines (except Psychiatry OPC) (Table 2). The average number of medicines IW, OPC, and combined was higher than the WHO optimal cut-off values (1.6–1.8) overall and across all the disciplines. The index for non-polypharmacy in all disciplines for IW, OPC, and combined was 0.30, 0.45, and 0.35, respectively, with values ranging from 0.85 at the Surgery OPC (highest) to 0.21 (lowest) in Clinical Medicine (OPC, IW, and Combined) (Table 3).

### 3.2. Prescribing Antibiotics

The percentage of encounters with an antibiotic in all disciplines was 26.4% (*n* = 328). It was significantly higher for IW than OPC prescriptions (44.0% vs. 9.8%) (*p* < 0.001). The highest percentage of encounters with an antibiotic was reported in Surgical IW prescriptions, which was 66.2% (*n* = 106), significantly higher than the respective IW values in all other disciplines (Table 2). There was no significant difference between disciplines with regard to OPC antibiotic prescribing. The highest combined percentage prescriptions with antibiotics was from Surgery (39.1%), followed by Clinical Medicine (31.2%) and Gynaecology and Obstetrics (21.2%). The percentage prescription with antibiotics was higher than the WHO optimal cut-off values (20–26.8%) for IW prescription in all disciplines, except Paediatrics; however, it was lower in OPC prescriptions across all disciplines. The index for rational antibiotic use in all disciplines for IW, OPC, and combined was 0.53, 1.00, and 0.89, respectively, with the lowest value being from Surgery IW prescriptions (0.35) (Table 3).

### 3.3. Encounters with an Injection

Percentage of encounters with an injection was 30.1% (*n* = 397), with a significantly higher percentage being reported from IW than OPC prescriptions (58.3% vs. 3.7%) (*p* < 0.001). Surgical IW prescriptions had the highest percentage of encounters with an injection (73.1%), and it was significantly higher than all other disciplines, except Clinical Medicine (Table 2). In the analysis of combined IW and OPC prescriptions, Clinical Medicine reported the highest percentage with an injection (43.4%), followed by Surgery (36.6%), Paediatrics (22.3%), Gynaecology and Obstetrics (20.9%), and Psychiatry (15.0%). The percentage prescription with injections was higher than the WHO optimal cut-off values (13.4–24.1%) for IW prescription in all disciplines, although it was lower for OPC prescriptions. In the combined analysis, only Clinical Medicine and Surgery had a higher percentage than the WHO optimal cut-off value. Index for safe injection use in all disciplines for IW, OPC, and combined was 0.32, 1.00, and 0.62, respectively, with the lowest value being for Surgery and Clinical Medicine IW prescriptions (0.26) (Table 3).

### 3.4. Generic Name Prescribing, Essential Medicines, and IRDP

Percentage of medicines prescribed by generic name was 90.1% (IW 89.5% vs. OPC 91.0%) in the combined analysis of all disciplines. The highest percentage of prescribing by generic name was identified from Psychiatry OPC prescriptions (99.1%), while the lowest percentage was from Paediatric OPC prescriptions (75.8%) (Table 2). The percentage of medicines prescribed by generic name was lower than the WHO optimal cut-off values (100%) across disciplines. Index of generic prescribing in all disciplines was 0.90, 0.91, and 0.90 for IW, OPC, and combined prescriptions, respectively (Table 3).

The overall percentage of medicines prescribed from the essential medicine list (EML) was 91.1% (IW 92.2% vs. OPC 89.7%). In the analysis of combined IW and OPC prescriptions, Surgery reported the highest percentage of EML medicines (96.3%), followed Gynaecology and Obstetrics (95.1%) and Clinical Medicine (90.4%). The percentage of EML medicines was lower than the WHO optimal cut-off values (100%) across disciplines. Index for EML prescribing in all disciplines for IW, OPC, and combined was 0.92, 0.90, and 0.91, respectively, with the lowest values being for prescriptions from Psychiatry and Surgery (0.73 and 0.86, respectively) (Table 3). The IRDP calculated for the different disciplines by adding the index values of all prescribing indicators (minimum 0; maximum 5) was highest for Gynaecology and Obstetrics (4.25), followed by Paediatrics (4.13) and Psychiatry (4.07). The lowest IRDP values were seen in the disciplines of Surgery (3.37) and Clinical Medicine (3.18). The overall IRDP for all disciplines was 3.67, with a significantly higher IRDP for OPC (4.26) than IW (2.97) (*p* < 0.001) (Table 3).

## 4. Discussion

To the best of our knowledge, the present study is the first comprehensive evaluation of core prescribing indicators at a tertiary care hospital in Sri Lanka, with comparisons made in practices in-ward and outpatient clinics across the 5 major disciplines. Since the South Asian region is home to nearly 1/4^th^ of the world's population, comprehensive evaluations of core prescribing indicators are important to understand current practices and promote the rational use of medicines in the region. To gauge a better understanding of the current finding in comparison with the regional and global context, we have compared the core prescribing indicators identified in the present study, with those of few developing countries from different regions of the world and in South Asia, with data arising from surveys conducted during similar time periods (Table 4); namely, these include countries from the African region (review) [7], Bangladesh [13], Brazil [14], China [15], India [16], Nepal [17], Pakistan [18], and UAE [19] (Table 4).

The average number of medicines per encounter in the current study was 4.8, which is 2–3 times higher than the WHO recommended value (1.6–1.8). This was similar for both IW and OPC prescriptions, across all disciplines. Clinical Medicine reported the highest values, which were more than 4 times higher than the WHO recommendations. In comparison with other countries (except Nepal), the average number of medicines per encounter in Sri Lanka was much higher, even when compared with studies from similar tertiary care health settings (Table 4). The high average number of medicines per prescription indicates a high degree of polypharmacy, generally considered when 4 or more medicines are prescribed per prescription. This could be due to recent changes in the epidemiological trend of diseases, with an increasing prevalence of noncommunicable diseases such as diabetes, hypertension, and coronary artery disease [20]. Furthermore, these diseases are often coexistent, which necessitates the simultaneous prescription of multiple medications for the same patient. Our results show that when disciplines of Paediatrics and Obstetrics were excluded, >50% of the present study population were >60 years of age, which also indicates the possibility of multiple comorbidities with the resultant increased in prescription of medicine. It is important to appreciate that polypharmacy in elderly is known to be associated with numerous negative health consequences [3]. Furthermore, polypharmacy in general can adversely influence treatment outcomes, reduce compliance, increase adverse effects, and lead to financial implications for the patient and national health care systems [21]. Hence, it is essential to implement local policies and practices to rationalizing the usage of medicines.

The overall usage of antibiotics in the present study (26.4%) was within the WHO recommended standard (20–26.8%). Furthermore, percentage of encounters with antibiotics were notably lower than most of the other countries compared, except Brazil, India, and UAE (Table 4). This indicates a positive trend towards reduction in indiscriminate use of antibiotics, especially at OPC. However, IW prescriptions (except Paediatrics) showed a higher percentage, which was 1.5–2 times higher than WHO recommendations. The higher usage of antibiotics IW could be due to the increased number of cases with infectious diseases. The study involved three large tertiary care health settings in Sri Lanka, which also serve as primary referral centers receiving patients from hospitals all over Sri Lanka. This probably also explains the higher than recommended percentage of encounters with injections (30.1%) observed in the present study, especially with regard to IW prescriptions (58.3%); however, antibiotics require more prudent prescribing, dispensing, and administration than other medicines because these medicines are at a greater risk of antimicrobial resistance [22]. Furthermore, an excessive use of injections instead of appropriate oral dosage forms available increases health care costs and may lead to higher probability of iatrogenic infections and adverse effects [18]. Hence, it is important to rationalize the IW usage of injectable medicines and antibiotics in the local study setting, by implementing guidelines and the judicious review of prescribing practices.

The WHO strongly advocates generic name prescribing because it helps to improve communication and clarity amongst health care providers and also helping to reduce medication costs for the patient [23]. Similarly, the concept of EML use is built on the premise that the use of a limited number of well-known and cost-effective medicines can lead to better health care, enhanced long-term medicines supply, and more equitable and sustainable access to products [23]. The percentages of medicines prescribed in generic name (90.1%) and from the EML (91.1%), although slightly lower than the WHO recommended standard (100%), were much higher in comparison with most other countries, except Pakistan (EML) and UAE (both) (Table 4). Literature evidence indicates that generic prescribing is better in public health care settings similar to the present study in comparison with private hospitals [7]. This is also observed in the Sri Lankan context with private sector surveys in 2002, indicating that only 36.7% of medicines are prescribed by generic name [24]. However, the recent National Medicines Regulatory Authority (NMRA) act of Sri Lanka (2015) implemented during the period of this study makes it compulsory for medical practitioners to write the generic name of a medicine in a prescription [25]. This change in policy would further help to improve the already acceptable levels of generic name prescribing noted in the government hospital settings in the present study.

Comparison of the different disciplines using the IRDP indicates that the disciplines of Gynaecology and Obstetrics, Paediatrics, and Psychiatry achieve acceptable levels of rational medication use (IRDP > 4), while further improvements are necessary in the disciplines of Clinical Medicine and Surgery. Our findings in the different disciplines will benefit policy makers in prioritizing and implementing service improvement strategies. The strengths of the present analysis include the comprehensive review of all prescriptions both IW and OPC, across the primary disciplines of medicine. Notable limitations include the lack of data in primary health care setting and private sector hospitals, reducing the generalizability of the results to all of Sri Lanka. Furthermore, although the core prescribing indicators are useful for investigating the medicines prescription pattern in primary care, they are less helpful for inpatient settings, and specialist outpatient facilities as in the present study, as medicines use patterns at these facilities are usually more complex [23]; however, our findings can be compared with similar studies performed in tertiary health care settings both within the national context and at a regional level. Seasonal variations in prescribing can impact on the prescribing indicators for a health facility, and the WHO recommends that data for prescribing should be collected over extended periods [23]; however, being a temperate country, with similar temperatures observed throughout the year, seasonal variations are unlikely to affect the prescribing patterns in the local setting.

## 5. Conclusions

In this study on prescribing indicators determined according to the WHO methodology, covering all 5 major disciplines both in the in-ward and outpatient settings in a tertiary care referral center in Sri Lanka, a high degree of polypharmacy was noted although the use of injectable medicines, EML medicines, and generic prescribing was satisfactory. The overall Index of Rational Drug Prescribing in this Sri Lankan tertiary care setting needs further improvement, as the setting is one of the main teaching hospitals in the country, where training of medical undergraduates and postgraduates take place. The list of most commonly prescribed medicines identified will be useful in determining the medicines, which should be given focus during teaching of medical students. Furthermore, investigation into the degree of rational prescribing associating it with clinical information in terms of prescriptions with polypharmacy will be important to further understand the problem of polypharmacy observed in this setting.

## Figures and Tables

**Table 1:** The ten most prescribed medicines in each discipline.

Number of prescriptions (%)				
Clinical Medicine (*N* = 320)	Obstetrics and Gynaecology (*N* = 320)	Paediatrics (*N* = 278)	Psychiatry (*N* = 80)	Surgery (*N* = 320)
Atorvastatin: 180 (56.2)	Folic acid 139 (44.3)	Salbutamol (In) 109 (39.2)	Benzhexol 30 (37.5)	Paracetamol: 133 (41.6)
Omeprazole: 129 (40.3)	Ferrous sulphate 129 (40.3)	Paracetamol 79 (28.4)	Olanzapine 30 (37.5)	Omeprazole: 104 (32.5)
Paracetamol: 115 (35.9)	Ascorbic acid 127 (39.7)	Beclomethasone inhaler 47 (16.9)	Risperidone 25 (31.2)	Thyroxine: 81 (25.3)
Aspirin: 105 (32.8)	Calcium lactate 111 (34.7)	Sodium valproate 46 (16.6)	Lorazepam 21 (26.2)	Diclofenac sodium: 76 (23.8)
Losartan: 91 (28.4)	Paracetamol 77 (24.1)	Chlorpheniramine 42 (15.1)	Clonazepam 16 (20.0)	Metronidazole: 56 (17.5)
Furosemide: 87 (27.2)	Metronidazole 52 (16.2)	Folic acid 33 (11.9)	Venlafaxine 16 (20.0)	Morphine (IV/SC): 51 (15.9)
Metformin: 84 (26.2)	Metoclopramide 44 (13.8)	Topiramate 28 (10.1)	Fluoxetine 13 (16.2)	Co-amoxiclav: 48 (15.0)
Clopidogrel: 83 (25.9)	Famotidine 39 (12.8)	Prednisolone 26 (9.4)	Lithium carbonate 10 (12.5)	Metoclopramide: 44 (13.8)
Salbutamol (In): 70 (21.9)	Diclofenac sodium 37 (11.6)	Carbamazepine 24 (8.6)	Lactulose 9 (11.2)	Cefuroxime: 42 (13.1)
Domperidone/Enalapril: 64^∗^ (20.0)	Domperidone 34 (10.6)	Domperidone 20 (7.2)	Promethazine (IM) 9 (11.2)	Domperidone 39 (12.8)

^∗^Equal number of prescriptions; IM, intramuscular; In, inhaled; IV, intravenous; SC, subcutaneous.

**Table 2:** In-ward, outpatient clinic, and combined prescribing indicators in the five disciplines.

Prescribing indicator (WHO recommended standard)	Mean ± SD/Number (%)					
	Clinical Medicine	Gynaecology and Obstetrics	Paediatrics	Psychiatry	Surgery	All disciplines
Average medicines per encounter (1.6–1.8)						
In-ward	7.8 ± 4.2 (8.5; 1–22)^∗^	4.3 ± 2.9 (4; 1–15)^∗†‡^	3.1 ± 2.6 (3; 1–18)^∗†‡^	6.2 ± 1.6 (4; 1–13)^†^	7.0 ± 4.0 (6.5; 1–19)^‡^	5.7 ± 4 (5; 1–22)
Outpatient clinic	7.8 ± 2.7 (7.5; 1–14)^∗†\^	3.2 ± 1.4 (4; 1–7)^∗^	2.6 ± 1.3 (2; 1–8)^∗^	2.7 ± 1.6 (2; 1–7)^†^	2.0 ± 1.0 (2; 1–6)^∗^	3.8 ± 2.8 (3; 1–14)
Both	7.8 ± 3.8 (7; 1–22)^∗†‡^	3.7 ± 2.4 (3; 1–15)^∗^	2.8 ± 1.9 (2; 1–18)^†‡^	4.4 ± 2.9 (3; 1–13)^†^	4.5 ± 3.8 (3; 1–19)^‡^	4.8 ± 3.6 (4; 1–22)

Encounters with an antibiotic (%) (20–26.8%)						
In-ward	85 (53.1)^∗†^	50 (31.2)^∗^	25 (21.2)^†‡^	15 (37.5)^‡^	106 (66.2)^∗‡^	281 (44.0)
Outpatient clinic	15 (9.4)	18 (11.2)	15 (9.4)	0	19 (11.9)	67 (9.8)
Both	100 (31.2)^∗‡^	68 (21.2)^∗^	40 (14.4)^∗^	15 (18.8)^‡^	125 (39.1)^∗‡^	348 (26.4)

Encounters with an injection (%) (13.4–24.1%)						
In-ward	116 (72.5)^∗†^	66 (41.2)^∗‡^	62 (52.5)^†#^	11 (27.5)^†#^	117 (73.1)^‡#^	372 (58.3)
Outpatient clinic	23 (14.4)^∗†^	1 (0.6)^∗^	0	1 (2.5)^†^	0	25 (3.7)
Both	139 (43.4)^∗†‡^	67 (20.9)^∗‡#^	62 (22.3)^†¥^	12 (15.0)	117 (36.6)^#¥^	397 (30.1)

Medicines prescribed in generic name (%) (100%)						
In-ward	1077 (86.4)^∗†‡^	625 (91.5)^∗^	328 (89.9)	230 (93.1)^†^	1018 (90.8)^‡^	3278 (89.5)
Outpatient clinic	1152 (92.1)^∗†‡^	492 (96.3)^∗^	319 (75.8)^†‡^	108 (99.1)^†^	310 (95.4%)^‡^	2381 (91.0)
Both	2229 (89.2)^∗†‡^	1117 (93.6)^∗^	647 (82.3)^†^	338 (94.9)^†^	1328 (91.8)^†^	5659 (90.1)

Medicines prescribed from EML (%) (100%)						
In-ward	1146 (91.9)^∗†^	649 (95.0)^∗^	316 (86.6)^∗†^	182 (73.7)^∗†^	1084 (96.7)^†^	3377 (92.2)
Outpatient clinic	1111 (88.8)^∗†^	487 (95.3)^∗†^	357 (84.8)^∗^	84 (77.1)^∗†^	308 (94.8)^†^	2347 (89.7)
Both	2257 (90.4)^∗†^	1136 (95.1)^∗^	673 (85.6)^∗†^	266 (72.9)^∗†^	1392 (96.3)^†^	5724 (91.1)

^∗†‡^Values in a single row with the same symbol are significantly different from one another; EML, essential medicines list; SD, standard deviation.

**Table 3:** Index of Rational Drug Prescribing (IRDP).


	Clinical Medicine	Gynaecology and Obstetrics	Paediatrics	Psychiatry	Surgery	All disciplines
Index of non-polypharmacy^∗^						
In-ward	0.21	0.39	0.55	0.27	0.24	0.30
Outpatient clinic	0.21	0.53	0.65	0.63	0.85	0.45
Both	0.21	0.46	0.61	0.39	0.38	0.35

Index of rational antibiotic use^†^						
In-ward	0.44	0.75	1.00	0.62	0.35	0.53
Outpatient clinic	1.00	1.00	1.00	—	1.00	1.00
Both	0.75	1.00	1.00	1.00	0.60	0.89

Index of safe injection use^‡^						
In-ward	0.26	0.46	0.36	0.68	0.26	0.32
Outpatient clinic	1.00	1.00	—	1.00	—	1.00
Both	0.43	0.90	0.84	1.00	0.51	0.62

Index of generic prescribing^#^						
In-ward	0.86	0.91	0.90	0.93	0.91	0.90
Outpatient clinic	0.92	0.96	0.76	0.99	0.95	0.91
Both	0.89	0.94	0.82	0.95	0.92	0.90

Index of EML prescribing^#^						
In-ward	0.92	0.95	0.87	0.74	0.97	0.92
Outpatient clinic	0.89	0.95	0.85	0.77	0.95	0.90
Both	0.90	0.95	0.86	0.73	0.96	0.91

Index of rational drug prescribing						
In-ward	2.69	3.46	3.68	3.24	2.73	2.97
Outpatient clinic	4.02	4.44	2.26	3.39	3.75	4.26
Both	3.18	4.25	4.13	4.07	3.37	3.67

Optimal value taken as ^∗^1.7, ^†^23.4, ^‡^18.75, and ^#^100; EML, essential medicines list.

**Table 4:** Comparison of core prescribing indictors in different countries.

Country (ref)	Core prescribing indictors (WHO recommended value)					
	Type of survey, year	Average medicines per encounter (1.6–1.8)	Encounters with an antibiotic (20–26.8) (%)	Encounters with an injection (13.4–24.1) (%)	Medicines prescribed in generic name (100%)	Medicines prescribed from EML (100%)
Sri Lanka∗	TCH, 2015	4.8	26.4	30.1	90.1%	91.1
Africa	Region (PC), 2006–2015	3.5	49.0	24.8	70.4%	88.9%
Bangladesh	TCH, 2010	3.6	48.0	1.3	1.3%	43.2%
Brazil	National, 2015	2.4	5.8	6.0	NR	45.1%
China	Provincial (PC), 2009–2010	3.2	50.9	24.4	NR	68.3%
India	TCH, 2018^#^	2.9	19.7	2.2	10.0%	22.6%
Nepal	TCH, 2016–2017	5.8	64.1	71.0	16.9%	47.6%
Pakistan	TCH, 2014–2015	2.8	51.5	0	56.6%	98.8%
UAE	TCH, 2012	2.5	9.8	3.1	100%	100%

^∗^Present study; ^#^year of publication; EML, essential medicines list; NR, not reported; PC, primary care; TCH, tertiary care hospital.

## Data Availability

Data can be obtained from the corresponding author upon request.

## Abbreviations

WHO: World Health Organization
INRUD: International Network of Rational Use of Drugs
GDP: Gross domestic product
NHSL: National Hospital of Sri Lanka
LRH: Lady Ridgeway Hospital for children
DSHW: De Soysa Hospital for Women
CM: Clinical Medicine
IRDP: Index of Rational Drug Prescribing
SPSS: Statistical Package for Social Sciences
IW: In-ward
OPC: Outpatient clinic
EML: Essential medicine list
NMRA: National Medicines Regulatory Authority.

## Supplementary Materials

Supplementary Materials Supplementary File 1: STROBE Statement—checklist of items that should be included in reports of cross-sectional studies. Supplementary Table S1: hundred most prescribed medicines in the five major specialties in ward and clinic settings in the tertiary care referral setting in Sri Lanka (*N* = 1322). Supplementary Table S2: Most commonly prescribed medicines in each specialty.
