# Supplementary material for: Core Prescribing Indicators and the Most Commonly Prescribed Medicines in a Tertiary Health Care Setting in a Developing Country
Source: Adv Pharmacol Pharm Sci. 2021 Jan 29;2021:6625377. doi: 10.1155/2021/6625377 (PMC7867447; doi:10.1155/2021/6625377)
Supplement: Supplementary Materials — Supplementary File 1: STROBE Statement—checklist of items that should be included in reports of cross-sectional studies. Supplementary Table S1: hundred most prescribed medicines in the five major specialties in ward and clinic settings in the tertiary care referral setting in Sri Lanka (N = 1322). Supplementary Table S2: Most commonly prescribed medicines in each specialty. [file 6625377.f1.zip › 6625377.f1/Table S1.docx]

**Table S1**: Hundred most prescribed medicines in the five major specialties in ward and clinic settings in the tertiary care referral setting in Sri Lanka (N=1322)

| Medication | Number of prescriptions | Percentage |
| --- | --- | --- |
| 1.     Paracetamol | 409 | 31.0 |
| 2.     Omeprazole | 272 | 20.6 |
| 3.     Folic acid | 241 | 18.3 |
| 4.     Atorvastatin | 213 | 16.2 |
| 5.     Salbutamol | 202 | 15.3 |
| 6.     Ferrous sulphate | 181 | 13.7 |
| 7.     Ascorbic acid | 171 | 13.0 |
| 8.     Calcium lactate | 166 | 12.6 |
| 9.     Domperidone | 159 | 12.1 |
| 10.   Aspirin | 150 | 11.4 |
| 11.   Metformin | 143 | 10.8 |
| 12.   Diclofenac sodium | 123 | 9.3 |
| 13.   Metronidazole | 121 | 9.2 |
| 14.   Thyroxine | 117 | 8.9 |
| 15.   Losartan | 114 | 8.6 |
| 16.   Metoclopramide | 110 | 8.3 |
| 17.   Co-amoxiclav | 106 | 8.0 |
| 18.   Furosemide | 97 | 7.4 |
| 19.   Cefuroxime | 92 | 7.0 |
| 20.   Beclomethasone inhaler | 89 | 6.8 |
| 21.   Clopidogrel | 89 | 6.8 |
| 22.   Enalapril | 84 | 6.4 |
| 23.   0.9% sodium chloride | 84 | 6.4 |
| 24.   Famotidine | 82 | 6.2 |
| 25.   Lactulose | 78 | 5.9 |
| 26.   Chlorpheniramine | 71 | 5.4 |
| 27.   Tramadol | 68 | 5.2 |
| 28.   Insulin-premixed (SC) | 67 | 5.1 |
| 29.   Sodium valproate | 66 | 5.0 |
| 30.   Prednisolone | 58 | 4.4 |
| 31.   1 α cholecalciferol | 58 | 4.4 |
| 32.   Morphine (SC/IV) | 57 | 4.3 |
| 33.   Atenolol | 54 | 4.1 |
| 34.   Vitamin B complex | 54 | 4.1 |
| 35.   Isosorbide mononitrate | 51 | 3.9 |
| 36.   Glyceryl tri nitrate sublingual | 47 | 3.6 |
| 37.   Prazosin | 47 | 3.6 |
| 38.   Calcium carbonate | 46 | 3.5 |
| 39.   Nifedipine | 44 | 3.3 |
| 40.   Amlodipine | 43 | 3.3 |
| 41.   Diazepam | 41 | 3.1 |
| 42.   Gliclazide | 38 | 2.9 |
| 43.   Hydrochlorothiazide | 38 | 2.9 |
| 44.   Tranexamic acid | 37 | 2.8 |
| 45.   Benzhexol | 36 | 2.7 |
| 46.   Ciprofloxacin | 36 | 2.7 |
| 47.   Clonazepam | 36 | 2.7 |
| 48.   Amitriptyline | 34 | 2.6 |
| 49.   Topiramate | 32 | 2.4 |
| 50.   Olanzapine | 31 | 2.4 |
| 51.   Spironolactone | 29 | 2.2 |
| 52.   Propantheline | 29 | 2.2 |
| 53.   Cloxacillin | 28 | 2.1 |
| 54.   Diltiazem | 28 | 2.1 |
| 55.   Tolbutamide | 28 | 2.1 |
| 56.   Carbamazepine | 27 | 2.0 |
| 57.   Ceftriaxone (IV) | 27 | 2.0 |
| 58.   Carvedilol | 26 | 2.0 |
| 59.   Gabapentin | 26 | 2.0 |
| 60.   Mefenamic acid | 26 | 2.0 |
| 61.   Risperidone | 26 | 2.0 |
| 62.   Amoxycillin | 24 | 1.8 |
| 63.   Promethazine (IM) | 24 | 1.8 |
| 64.   Ranitidine | 24 | 1.8 |
| 65.   Captopril | 23 | 1.7 |
| 66.   Ipratropium (inhalation) | 23 | 1.7 |
| 67.   Penicillin C | 23 | 1.7 |
| 68.   Enoxaparin (SC) | 22 | 1.7 |
| 69.   Hydrocortisone | 21 | 1.6 |
| 70.   Lorazepam | 21 | 1.6 |
| 71.   Clobazam | 20 | 1.5 |
| 72.   Clarithromycin | 19 | 1.4 |
| 73.   Pethidine | 18 | 1.4 |
| 74.   Venlafaxine | 18 | 1.4 |
| 75.   Dextrose (IV) | 18 | 1.4 |
| 76.   Norethisterone | 17 | 1.3 |
| 77.   Theophylline | 17 | 1.3 |
| 78.   Voriconazole | 17 | 1.3 |
| 79.   Vitamin K | 17 | 1.3 |
| 80.   Bisacodyl | 16 | 1.2 |
| 81.   Cephalexin | 16 | 1.2 |
| 82.   Cotrimoxazole | 16 | 1.2 |
| 83.   Meropenem | 16 | 1.2 |
| 84.   Thiamine | 15 | 1.1 |
| 85.   Fluoxetine | 14 | 1.1 |
| 86.   Propranolol | 14 | 1.1 |
| 87.   Lamotrigine | 13 | 1.0 |
| 88.   Methyldopa | 13 | 1.0 |
| 89.   Methyl salicylate | 13 | 1.0 |
| 90.   Thriposha | 13 | 1.0 |
| 91.   Sulphasalazine | 13 | 1.0 |
| 92.   Glibenclamide | 12 | 0.9 |
| 93.   Calcium gluconate (IV) | 11 | 0.8 |
| 94.   Doxycycline | 11 | 0.8 |
| 95.   Potassium chloride | 11 | 0.8 |
| 96.   Lithium carbonate | 11 | 0.8 |
| 97.   Dexamethasone | 10 | 0.8 |
| 98.   Sodium bicarbonate | 10 | 0.8 |
| 99.   Tamsulosin | 10 | 0.8 |

IM – Intramuscular; IV – Intravenous; SC – Subcutaneous;
