# Supplementary material for: Core Prescribing Indicators and the Most Commonly Prescribed Medicines in a Tertiary Health Care Setting in a Developing Country
Source: Adv Pharmacol Pharm Sci. 2021 Jan 29;2021:6625377. doi: 10.1155/2021/6625377 (PMC7867447; doi:10.1155/2021/6625377)
Supplement: Supplementary Materials — Supplementary File 1: STROBE Statement—checklist of items that should be included in reports of cross-sectional studies. Supplementary Table S1: hundred most prescribed medicines in the five major specialties in ward and clinic settings in the tertiary care referral setting in Sri Lanka (N = 1322). Supplementary Table S2: Most commonly prescribed medicines in each specialty. [file 6625377.f1.zip › 6625377.f1/Table S2.docx]

**Table S2: Most commonly prescribed medicines in each specialty**

| Number of Prescriptions (%) | | | | | | | | | |
| --- | --- | --- | --- | --- | --- | --- | --- | --- | --- |
| Clinical Medicine | | Gynaecology and Obstetrics | | Paediatrics | | Psychiatry | | Surgery | |
|  |  |  |  |  |  |  |  |  |  |
| 1. Atorvastatin | 180 (56.3) | 1. Folic acid | 139 (43.4) | 1. Salbutamol | 109 (39.2) | 1. Benzhexol | 30 (37.5) | 1. Paracetamol | 133 (41.6) |
| 1. Omeprazole | 129 (40.3) | 1. Ferrous sulphate | 129 (40.3) | 1. Paracetamol | 79 (28.4) | 1. Olanzapine | 30 (37.5) | 1. Omeprazole | 104 (32.5) |
| 1. Paracetamol | 115 (35.9) | 1. Ascorbic acid | 127 (39.7) | 1. Beclomethasone inhaler | 47 (16.9) | 1. Risperidone | 25 (31.3) | 1. Thyroxine | 81 (25.3) |
| 1. Aspirin | 105 (32.8) | 1. Calcium lactate | 111 (34.7) | 1. Sodium valproate | 46 (16.5) | 1. Lorazepam | 21 (26.3) | 1. Diclofenac sodium | 76 (23.8) |
| 1. Losartan | 91 (28.4) | 1. Paracetamol | 77 (24.1) | 1. Chlorpheniramine | 42 (15.1) | 1. Clonazepam | 16 (20) | 1. Metronidazole | 56 (17.5) |
| 1. Furosemide | 87 (27.2) | 1. Metronidazole | 52 (16.3) | 1. Folic acid | 33 (11.9) | 1. Venlafaxine | 16 (20) | 1. Morphine (IV/SC) | 51 (15.9) |
| 1. Metformin | 84 (26.3) | 1. Metoclopramide | 44 (13.8) | 1. Topiramate | 28 (10.1) | 1. Fluoxetine | 13 (16.3) | 1. Co-amoxiclav | 48 (15) |
| 1. Clopidogrel | 83 (25.9) | 1. Famotidine | 39 (12.2) | 1. Prednisolone | 26 (9.4) | 1. Lithium carbonate | 10 (12.5) | 1. Metoclopramide | 44 (13.8) |
| 1. Salbutamol | 70 (21.9) | 1. Diclofenac sodium | 37 (11.6) | 1. Carbamazepine | 24 (8.6) | 1. Lactulose | 9 (11.3) | 1. Cefuroxime | 42 (13.1) |
| 1. Domperidone | 64 (20) | 1. Domperidone | 34 (10.6) | 1. Domperidone | 20 (7.2) | 1. Promethazine (IM) | 9 (11.3) | 1. Domperidone | 39 (12.2) |
| 1. Enalapril | 64 (20) | 1. Cefuroxime | 32 (10) | 1. Clobazam | 19 (6.8) | 1. Metformin | 8 (10) | 1. Famotidine | 35 (10.9) |
| 1. Isosorbide mononitrate | 49 (15.3) | 1. Tramadol | 26 (8.1) | 1. Clonazepam | 17 (6.1) | 1. Diazepam | 7 (8.8) | 1. Lactulose | 34 (10.6) |
| 1. Glyceryl trinitrate (SL) | 46 (14.4) | 1. Omeprazole | 25 (7.8) | 1. 0.9% Sodium chloride | 16 (5.8) | 1. Haloperidol | 7 (8.8) | 1. 1 α cholecalciferol | 32 (10) |
| 1. Atenolol | 45 (14.1) | 1. Mefenamic acid | 24 (7.5) | 1. Ascorbic acid | 14 (5) | 1. Sertraline | 7 (8.8) | 1. Calcium lactate | 32 (10) |
| 1. Insulin (SC) | 41 (12.8) | 1. Metformin | 24 (7.5) | 1. Co-amoxiclav | 13 (4.7) | 1. Co-amoxiclav | 6 (7.5) | 1. Folic acid | 29 (9.1) |
| 1. Folic acid | 40 (12.5) | 1. Tranexamic acid | 24 (7.5) | 1. Thriposha | 13 (4.7) | 1. Imipramine | 6 (7.5) | 1. Atorvastatin | 28 (8.8) |
| 1. Amlodipine | 39 (12.2) | 1. Aspirin | 21 (6.6) | 1. Ferrous sulphate | 13 (4.7) | 1. Sodium valproate | 6 (7.5) | 1. Tramadol | 28 (8.8) |
| 1. Beclomethasone | 39 (12.2) | 1. Norethisterone | 17 (5.3) | 1. Omeprazole | 12 (4.3) | 1. Thyroxine | 6 (7.5) | 1. 0.9% sodium chloride | 27 (8.4) |
| 1. Prazosin | 39 (12.2) | 1. Amoxycillin | 13 (4.1) | 1. Hydrocortisone | 9 (3.2) | 1. Enalapril | 5 (6.3) | 1. Diazepam | 25 (7.8) |
| 1. 0.9% sodium chloride (IV) | 35 (10.9) | 1. Pethidine | 11 (3.4) | 1. Lamotrigine | 9 (3.2) | 1. Paracetamol | 5 (6.3) | 1. Metformin | 25 (7.8) |
| 1. Vit B complex | 33 (10.3) | 1. Co-amoxiclav | 9 (2.8) | 1. Amoxycillin | 7 (2.5) | 1. Thiamine | 5 (6.3) | 1. Aspirin | 20 (6.3) |
| 1. Co-amoxiclav | 30 (9.4) | 1. Doxycycline | 9 (2.8) | 1. Lactulose | 7 (2.5) | 1. Chlorpromazine | 4 (5) | 1. Salbutamol | 20 (6.3) |
| 1. Gliclazide | 30 (9.4) | 1. Promethazine (IM) | 9 (2.8) | 1. Benzhexol | 6 (2.2) | 1. Clopidogrel | 4 (5) | 1. Calcium carbonate | 19 (5.9) |
| 1. Hydrochlorothiazide | 28 (8.8) | 1. Ranitidine | 9 (2.8) | 1. Fludrocortisone/Salmeterol (In) | 6 (2.2) | 1. Famotidine | 4 (5) | 1. Losartan | 18 (5.6) |
| 1. Methylsalicylate | 27 (8.4) | 1. Conjugated oestrogen | 8 (2.5) | 1. Miconazole | 6 (2.2) | 1. Lamotrigine | 4 (5) | 1. Insulin (SC) | 16 (5) |
| 1. Amitriptyline | 25 (7.8) | 1. Insulin (SC) | 8 (2.5) | 1. Calcium lactate | 5 (1.8) | 1. Propranolol | 4 (5) | 1. Penicillin C | 16 (5) |
| 1. Nifedipine | 25 (7.8) | 1. Cephalexin | 7 (2.2) | 1. Cephalexin | 5 (1.8) | 1. Trifluoperazine | 4 (5) | 1. Ciprofloxacin | 15 (4.7) |
| 1. Ferrous sulphate | 24 (7.5) | 1. Ciprofloxacin | 6 (1.9) | 1. Dextrose (IV) | 5 (1.8) | 1. Chlorpheniramine | 3 (3.8) | 1. Enalapril | 14 (4.4) |
| 1. Spironolactone | 24 (7.5) | 1. Dexamethasone | 6 (1.9) | 1. Thyroxine | 5 (1.8) | 1. Clozapine | 3 (3.8) | 1. Ferrous sulphate | 14 (4.4) |
| 1. Ceftriaxone (IV) | 23 (7.2) | 1. Lactulose | 6 (1.9) | 1. Ampicillin (IV) | 4 (1.4) | 1. Gliclazide | 3 (3.8) | 1. Vit B complex | 13 (4.1) |
| 1. Thyroxine | 23 (7.2) | 1. Methyldopa | 6 (1.9) | 1. Cefotaxime (IV) | 4 (1.4) | 1. Mirtazapine | 3 (3.8) | 1. Sulphasalazine | 12 (3.8) |
| 1. Calcium carbonate | 22 (6.9) | 1. Diazepam | 5 (1.6) | 1. Chloral hydrate | 4 (1.4) | 1. Nifedipine | 3 (3.8) | 1. Ranitidine | 11 (3.4) |
| 1. Carvedilol | 22 (6.9) | 1. Nifedipine | 5 (1.6) | 1. Furosemide | 4 (1.4) | 1. Quetiapine | 3 (3.8) | 1. Ascorbic acid | 10 (3.1) |
| 1. Lactulose | 22 (6.9) | 1. 0.9% Sodium chloride | 5 (1.6) | 1. Penicillin C | 4 (1.4) | 1. Alprazolam | 2 (2.5) | 1. Cloxacillin | 10 (3.1) |
| 1. Diltiazem | 21 (6.6) | 1. Aluminium hydroxide | 4 (1.3) | 1. Baclofen | 4 (1.4) | 1. Amisulpride | 2 (2.5) | 1. Nifedipine | 10 (3.1) |
| 1. Metoclopramide | 21 (6.6) | 1. Chlorpheniramine | 4 (1.3) | 1. Zinc sulphate | 4 (1.4) | 1. Aripiprazole | 2 (2.5) | 1. Vitamin K | 10 (3.1) |
| 1. Prednisolone | 21 (6.6) | 1. Cloxacillin | 4 (1.3) | 1. Cloxacillin | 3 (1.1) | 1. Ascorbic acid | 2 (2.5) | 1. Amitriptyline | 9 (2.8) |
| 1. Gabapentin | 20 (6.3) | 1. Doxambucil | 4 (1.3) | 1. Phenobarbitone | 3 (1.1) | 1. Aspirin | 2 (2.5) | 1. Tolbutamide | 9 (2.8) |
| 1. 1 α cholecalciferol | 20 (6.3) | 1. Erythromycin | 4 (1.3) | 1. Theophylline | 3 (1.1) | 1. Atenolol | 2 (2.5) | 1. Bisacodyl | 8 (2.5) |
| 1. Tolbutamide | 19 (5.9) | 1. Glibenclamide | 4 (1.3) | 1. 1 α cholecalciferol | 2 (0.7) | 1. Atorvastatin | 2 (2.5) | 1. Chlorpheniramine | 8 (2.5) |
| 1. Ascorbic acid | 18 (5.6) | 1. Losartan | 4 (1.3) | 1. Aspirin | 2 (0.7) | 1. Azathioprine | 2 (2.5) | 1. Prednisolone | 8 (2.5) |
| 1. Captopril | 18 (5.6) | 1. Oral contraceptive pill | 4 (1.3) | 1. Azithromycin | 2 (0.7) | 1. Cephalexin | 2 (2.5) | 1. Enoxaparin (SC) | 7 (2.2) |
| 1. Calcium lactate | 17 (5.3) | 1. Vitamin B complex | 4 (1.3) | 1. Probiotics | 2 (0.7) | 1. Domperidone | 2 (2.5) | 1. Hydrochlorothiazide | 7 (2.2) |
| 1. Ipratropium (inhalation) | 17 (5.3) | 1. Atorvastatin | 3 (0.9) | 1. Calcium carbonate | 2 (0.7) | 1. Fluphenazine | 2 (2.5) | 1. Prazosin | 7 (2.2) |
| 1. Cefuroxime | 16 (5) | 1. Bisacodyl | 3 (0.9) | 1. Clarithromycin | 2 (0.7) | 1. Insulin (SC) | 2 (2.5) | 1. Atenolol | 6 (1.9) |
| 1. Clarithromycin | 16 (5) | 1. Clomiphene citrate | 3 (0.9) | 1. Famotidine | 2 (0.7) | 1. Omeprazole | 2 (2.5) | 1. Dextrose (IV) | 6 (1.9) |
| 1. Meropenem (IV) | 16 (5) | 1. Oxytocin (IV) | 3 (0.9) | 1. Immunoglobulin (IV) | 2 (0.7) | 1. Prednisolone | 2 (2.5) | 1. Gabapentin | 6 (1.9) |
| 1. Chlorpheniramine | 14 (4.4) | 1. Vitamin B12 (IM) | 3 (0.9) | 1. Mebendazole | 2 (0.7) | 1. Salbutamol | 2 (2.5) | 1. Hydrocortisone | 6 (1.9) |
| 1. Ciprofloxacin | 14 (4.4) | 1. Hyoscine butylbromide | 3 (0.9) | 1. Metformin | 2 (0.7) | 1. Vitamin B complex | 2 (2.5) | 1. Clindamycin | 5 (1.6) |
| 1. Tramadol | 14 (4.4) | 1. Calcium carbonate | 2 (0.6) | 1. Methylprednisolone (IV) | 2 (0.7) | 1. 1 α cholecalciferol | 1 (1.3) | 1. Diltiazem | 5 (1.6) |
| 1. Enoxaparin (SC) | 13 (4.1) | 1. Enoxaparin (SC) | 2 (0.6) | 1. Phenytoin Na | 2 (0.7) | 1. Activated charcoal | 1 (1.3) | 1. Gliclazide | 5 (1.6) |
| 1. Na valproate | 13 (4.1) | 1. Hydrochlorothiazide | 2 (0.6) | 1. Terbutaline | 2 (0.7) | 1. Aluminium hydroxide | 1 (1.3) | 1. Potassium chloride | 5 (1.6) |
| 1. Tranexamic acid | 12 (3.8) | 1. Mebendazole | 2 (0.6) | 1. Vitamin B complex | 2 (0.7) | 1. Amlodipine | 1 (1.3) | 1. Propranolol | 5 (1.6) |
| 1. Cotrimoxazole | 11 (3.4) | 1. Morphine (SC) | 2 (0.6) | 1. Multivitamin | 2 (0.7) | 1. Bisoprolol | 1 (1.3) | 1. Tamsulosin | 5 (1.6) |
| 1. Metronidazole | 11 (3.4) | 1. Nimodipine | 2 (0.6) | 1. Vigabatrin | 2 (0.7) | 1. Bisacodyl | 1 (1.3) | 1. Terbutaline | 5 (1.6) |
| 1. Nicorandil | 11 (3.4) | 1. Ondansetron (IV) | 2 (0.6) | 1. Phosphate buffer solution | 2 (0.7) | 1. Beclomethasone | 1 (1.3) | 1. Calcium gluconate (IV) | 4 (1.3) |
| 1. Cloxacillin | 10 (3.1) | 1. Pantoprazole | 2 (0.6) | 1. Acyclovir | 1 (0.4) | 1. Calcium carbonate | 1 (1.3) | 1. Carvedilol | 4 (1.3) |
| 1. Diclofenac Na | 10 (3.1) | 1. Prostaglandin vaginal tablets | 2 (0.6) | 1. Betamethasone | 1 (0.4) | 1. Calcium lactate | 1 (1.3) | 1. Cotrimoxazole | 4 (1.3) |
| 1. Sodium bicarbonate | 9 (2.8) | 1. Thyroxine | 2 (0.6) | 1. Bisacodyl | 1 (0.4) | 1. Captopril | 1 (1.3) | 1. Furosemide | 4 (1.3) |
| 1. Prochlorperazine | 8 (2.5) | 1. Acyclovir | 1 (0.3) | 1. Bromocriptine | 1 (0.4) | 1. Cefuroxime | 1 (1.3) | 1. Ipratropium (In) | 4 (1.3) |
| 1. Theophylline | 8 (2.5) | 1. Ampicillin (IV) | 1 (0.3) | 1. Captopril | 1 (0.4) | 1. Chlordiazepoxide | 1 (1.3) | 1. Nimodipine | 4 (1.3) |
| 1. Calcium resonium | 7 (2.2) | 1. Atenolol | 1 (0.3) | 1. Ceftriaxone (IV) | 1 (0.4) | 1. Ciprofloxacin | 1 (1.3) | 1. Ondansetron (IV) | 4 (1.3) |
| 1. Calcium gluconate (IV) | 7 (2.2) | 1. Azithromycin | 1 (0.3) | 1. Cefuroxime | 1 (0.4) | 1. Cloxacillin | 1 (1.3) | 1. Pethidine | 4 (1.3) |
| 1. Erythropoietin | 7 (2.2) | 1. Povidone iodine vaginal pessary | 1 (0.3) | 1. Cetirizine | 1 (0.4) | 1. Cremaffin | 1 (1.3) | 1. Theophylline | 4 (1.3) |
| 1. Pioglitazone | 7 (2.2) | 1. Carbamazepine | 1 (0.3) | 1. Clonidine | 1 (0.4) | 1. Diltiazem | 1 (1.3) | 1. Thiamine | 4 (1.3) |
| 1. Dextrose (IV) | 7 (2.2) | 1. Diltiazem | 1 (0.3) | 1. Cotrimoxazole | 1 (0.4) | 1. Flunarizine | 1 (1.3) | 1. Amlodipine | 3 (0.9) |
| 1. Hydrocortisone | 6 (1.9) | 1. Enalapril | 1 (0.3) | 1. Deferoxamine (IV) | 1 (0.4) | 1. Furosemide | 1 (1.3) | 1. Captopril | 3 (0.9) |
| 1. Isosorbide dinitrate | 6 (1.9) | 1. Furosemide | 1 (0.3) | 1. Deferasirox | 1 (0.4) | 1. Glipizide | 1 (1.3) | 1. Carbimazole | 3 (0.9) |
| 1. Methyldopa | 6 (1.9) | 1. Gentamicin (IV) | 1 (0.3) | 1. Erythropoietin (SC) | 1 (0.4) | 1. Hydrochlorothiazide | 1 (1.3) | 1. Ceftriaxone (IV) | 3 (0.9) |
| 1. Phenytoin Na | 6 (1.9) | 1. Ibuprofen | 1 (0.3) | 1. Flucloxacillin | 1 (0.4) | 1. Imidapril | 1 (1.3) | 1. Glibenclamide | 3 (0.9) |
| 1. Vitamin K | 6 (1.9) | 1. Ipratropium (inhalation) | 1 (0.3) | 1. Fluoxetine | 1 (0.4) | 1. Ipratropium (inhalation) | 1 (1.3) | 1. Lignocaine | 3 (0.9) |
| 1. Allopurinol | 5 (1.6) | 1. Isosorbide mononitrate | 1 (0.3) | 1. Flunarizine | 1 (0.4) | 1. Isosorbide dinitrate | 1 (1.3) | 1. Promethazine (IM) | 3 (0.9) |
| 1. Glibenclamide | 5 (1.6) | 1. Labetalol | 1 (0.3) | 1. Gentamicin (IV) | 1 (0.4) | 1. Losartan | 1 (1.3) | 1. Spironolactone | 3 (0.9) |
| 1. Potassium chloride | 5 (1.6) | 1. Misoprostol | 1 (0.3) | 1. Goserelin (SC) | 1 (0.4) | 1. Metoclopramide | 1 (1.3) | 1. Vitamin D | 3 (0.9) |
| 1. Sodium chloride | 5 (1.6) | 1. Polybion (IV) | 1 (0.3) | 1. Human albumin (IV) | 1 (0.4) | 1. Metronidazole | 1 (1.3) | 1. Amoxycillin | 2 (0.6) |
| 1. Propranolol | 5 (1.6) | 1. Prednisolone | 1 (0.3) | 1. Hydroxychloroquine | 1 (0.4) | 1. Midazolam (IV) | 1 (1.3) | 1. Betahistine | 2 (0.6) |
| 1. Salmeterol/Fluticasone (In) | 5 (1.6) | 1. Progesterone | 1 (0.3) | 1. Ketamine (IV) | 1 (0.4) | 1. Sodium chloride | 1 (1.3) | 1. Beclomethasone | 2 (0.6) |
| 1. Sitagliptin | 5 (1.6) | 1. Salbutamol | 1 (0.3) | 1. Potassium chloride | 1 (0.4) | 1. 0.9% Sodium chloride | 1 (1.3) | 1. Ceftazidime (IV) | 2 (0.6) |
| 1. Tamsulosin | 5 (1.6) | 1. Theophylline | 1 (0.3) | 1. Levamisole | 1 (0.4) | 1. Paroxetine | 1 (1.3) | 1. Clopidogrel | 2 (0.6) |
| 1. Thiamine | 5 (1.6) | 1. Thiamine | 1 (0.3) | 1. Metolazone | 1 (0.4) | 1. Ranitidine | 1 (1.3) | 1. Dexamethasone | 2 (0.6) |
| 1. Diazepam | 4 (1.3) | 1. Warfarin | 1 (0.3) | 1. Metronidazole | 1 (0.4) | 1. Spironolactone | 1 (1.3) | 1. Erythromycin | 2 (0.6) |
| 1. Digoxin | 4 (1.3) |  |  | 1. Midazolam (IV) | 1 (0.4) | 1. Theophylline | 1 (1.3) | 1. Human albumin (IV) | 2 (0.6) |
| 1. Dipyridamole | 4 (1.3) |  |  | 1. Mupirocin | 1 (0.4) | 1. Clomipramine | 1 (1.3) | 1. Imipenem (IV) | 2 (0.6) |
| 1. Metolazone | 4 (1.3) |  |  | 1. Sodium chloride | 1 (0.4) |  |  | 1. Mefenamic acid | 2 (0.6) |
| 1. Morphine (SC) | 4 (1.3) |  |  | 1. Nifedipine | 1 (0.4) |  |  | 1. Phenoxybenzamine | 2 (0.6) |
| 1. Miconazole | 4 (1.3) |  |  | 1. Polycitrate | 1 (0.4) |  |  | 1. Sitagliptin | 2 (0.6) |
| 1. Aluminium hydroxide | 3 (0.9) |  |  | 1. Prazosin | 1 (0.4) |  |  | 1. Methyl salicylate | 2 (0.6) |
| 1. Betamethasone | 3 (0.9) |  |  | 1. Ranitidine (IV) | 1 (0.4) |  |  | 1. Acyclovir | 1 (0.3) |
| 1. Bisacodyl | 3 (0.9) |  |  | 1. Risperidone | 1 (0.4) |  |  | 1. Allopurinol | 1 (0.3) |
| 1. Carbimazole | 3 (0.9) |  |  | 1. Salmeterol/ Fluticasone (In) | 1 (0.4) |  |  | 1. Amikacin (IV) | 1 (0.3) |
| 1. Ceftazidime (IV) | 3 (0.9) |  |  | 1. Simethicone | 1 (0.4) |  |  | 1. Ampicillin (IV) | 1 (0.3) |
| 1. Cetirizine | 3 (0.9) |  |  | 1. Sodium benzoate | 1 (0.4) |  |  | 1. Azathioprine | 1 (0.3) |
| 1. Clonazepam | 3 (0.9) |  |  | 1. Spironolactone | 1 (0.4) |  |  | 1. Hyoscine butylbromide | 1 (0.3) |
| 1. Fluconazole | 3 (0.9) |  |  | 1. Vitamin A | 1 (0.4) |  |  | 1. Calcium resonium | 1 (0.3) |
| 1. Human albumin (IV) | 3 (0.9) |  |  | 1. Vitamin D | 1 (0.4) |  |  | 1. Cefotaxime (IV) | 1 (0.3) |
| 1. Methotrexate | 3 (0.9) |  |  | 1. Vitamin E | 1 (0.4) |  |  | 1. Chlordiazepoxide | 1 (0.3) |
| 1. Mycophenolate mofetil | 3 (0.9) |  |  | 1. Vitamin K | 1 (0.4) |  |  | 1. Clarithromycin | 1 (0.3) |
| 1. Nimodipine | 3 (0.9) |  |  | 1. Sulphasalazine | 1 (0.4) |  |  | 1. Doxambucil | 1 (0.3) |
| 1. Pantoprazole | 3 (0.9) |  |  | 1. Aqueous cream | 1 (0.4) |  |  | 1. Doxycycline | 1 (0.3) |
| 1. Phenoxymethyl penicillin | 3 (0.9) |  |  | 1. Deriphylline | 1 (0.4) |  |  | 1. Furazolidone | 1 (0.3) |

IM – intramuscular; In – inhaler; IV – intravenous; SC – subcutaneous; SL – sub-lingual
